# Enhanced accessibility and hydrophobicity of amyloidogenic intermediates of the β2-microglobulin D76N mutant revealed by high-pressure experiments

**DOI:** 10.1016/j.jbc.2021.100333

**Published:** 2021-01-26

**Authors:** Kazumasa Sakurai, Ryosuke Tomiyama

**Affiliations:** 1 High Pressure Protein Research Center, Institute of Advanced Technology, Kindai University, Wakayama, Japan; 2 Department of Biotechnology, Faculty of Biology-oriented Science and Technology, Kindai University, Wakayama, Japan

**Keywords:** amyloid, protein folding, mutant, biophysics, fluorescence, high-pressure experiment, β2-microglobulin, <ν>, center of the spectral mass, β2m, β2-microglobulin, ANS, 1-anilino-8-naphthalene sulfonate, MD, molecular dynamics, MSA, molecular surface areas

## Abstract

β2-Microglobulin (β2m) is the causative protein of dialysis-related amyloidosis. Its unfolding mainly proceeds along the pathway of N_C_ →U_C_ ⇄ U_T_, whereas refolding follows the U_T_ → I_T_ (→N_T_) →N_C_ pathway, in which N, I, and U are the native, intermediate, and unfolded states, respectively, with the Pro32 peptidyl-prolyl bond in *cis* or *trans* conformation as indicated by the subscript. It is noted that the I_T_ state is a putative amyloidogenic precursor state. Several aggregation-prone variants of β2m have been reported to date. One of these variants is D76N β2m, which is a naturally occurring amyloidogenic mutant. To elucidate the molecular mechanisms contributing to the enhanced amyloidogenicity of the mutant, we investigated the equilibrium and kinetic transitions of pressure-induced folding/unfolding equilibria in the wild type and D76N mutant by monitoring intrinsic tryptophan and 1-anilino-8-naphthalene sulfonate fluorescence. An analysis of kinetic data revealed that the different folding/unfolding behaviors of the wild type and D76N mutant were due to differences in the activation energy between the unfolded and the intermediate states as well as stability of the native state, leading to more rapid accumulation of I_T_ state for D76N in the refolding process. In addition, the I_T_ state was found to assume more hydrophobic nature. These changes induced the enhanced amyloidogenicity of the D76N mutant and the distinct pathogenic symptoms of patients. Our results suggest that the stabilization of the native state will be an effective approach for suppressing amyloid fibril formation of this mutant.

β2-Microglobulin (β2m) is a light chain of the type I major histocompatibility antigen (MHC-1) with 99 amino acid residues. β2m consists of 7 β-strands (A-G). Strands A, B, E, and D form one β-sheet, while strands C, F, and G form another sheet, with a disulfide bond between Cys25 and Cys80 linking the two sheets (Fig. 1) (1). It is the causative protein of dialysis-related amyloidosis, a serious complication in patients receiving hemodialysis for more than 10 years (2, 3). In the last few decades, the structural properties of β2m amyloid fibrils and the physicochemical mechanisms of fibril formation have been extensively examined (2, 4, 5, 6, 7).

**Figure 1:**
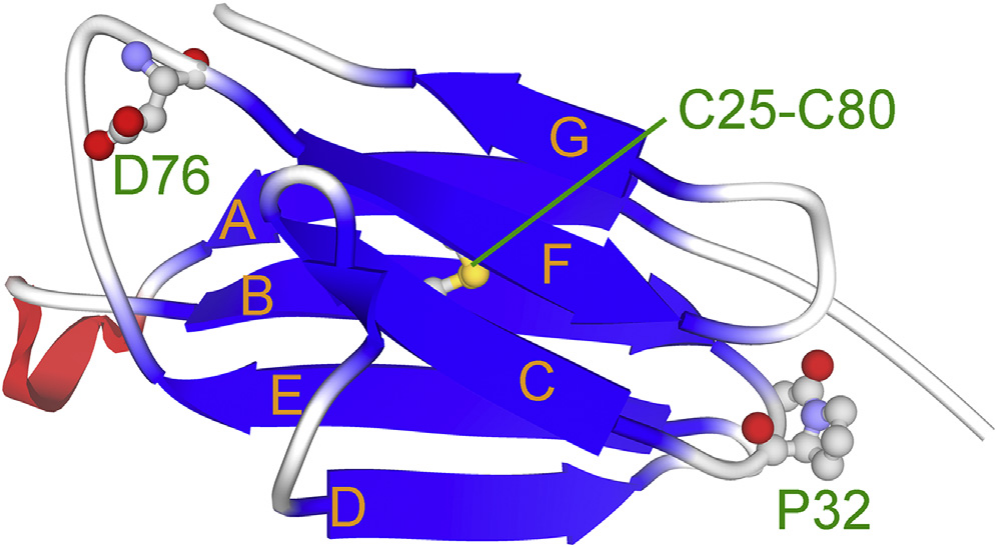
**Crystal structures ofβ2m(PDB ID: 2yxf** (1)**).** The side chains of P32, D76, C25, and C80 are depicted as *balls* and *sticks*.

The role of the folding/unfolding process in amyloidogenicity has also been investigated. Sakata *et al.* (8) and Jahn *et al.* (9) independently examined denaturant-induced unfolding and refolding using a stopped-flow apparatus and proposed mechanisms for the folding of β2m. Although differences were noted in the number of states appearing in the folding pathway as well as rate constants between these states, a common feature was that the pathway assumed an annular scheme and was composed of fast and slow steps. The ends of the folding/unfolding reactions of β2m are the N_C_ and U_T_ states, where N_C_ is the native state with Pro32 in the *cis* conformation (Fig. 1) and U_T_ is the unfolded state with Pro32 in the *trans* conformation. Slow steps are associated with the *cis*-*trans* isomerization of the peptidyl-prolyl bond at Pro32. Due to slow *cis*-*trans* isomerization processes, the unfolding of β2m mainly proceeds along the N_C_ → U_C_ ⇄ U_T_ pathway, whereas that of refolding follows the U_T_ → I_T_ (→N_T_) →N_C_ pathway. It is important to note that the I_T_ state, which mainly occurs in the refolding process, has been proposed as an amyloidogenic precursor state (10).

Several aggregation-prone variants of β2m have been reported to date. One of these variants is D76N β2m, which is a naturally occurring amyloidogenic mutant. D76N β2m was discovered in the members of a French family who had progressive bowel dysfunction with extensive visceral amyloid deposits composed of β2m (11). In contrast to patients with dialysis-related amyloidosis, all members of this family had normal circulating concentrations of β2m and none of the osteoarticular deposits characteristic of dialysis-related amyloidosis. Mangione *et al.* (12) reported that fibril formation by D76N β2m was primed by an exposure to a hydrophobic–hydrophilic interface under physiological intensity shear flow. In addition, wild-type β2m was recruited by the mutant into amyloid fibrils *in vitro*, but was absent from amyloid deposited *in vivo* (12).

Although the mechanisms contributing to the enhanced amyloidogenicity of D76N have been investigated, they have not yet been elucidated in detail. Chong *et al.* (13) performed a molecular dynamics (MD) simulation of the I_T_ state to examine its structural properties. The findings obtained revealed the more hydrophobic nature of D76N than the wild type; however, the D76N I_T_ state assumed a more compact, structured state. We also examined differences in the properties of the native states between wild-type and D76N β2ms using high-pressure NMR measurements and an MD simulation (14). The findings obtained revealed that the peripheral region, including the C and D strands, of D76N was less flexible than that of the wild type. However, this consolidation induced the loosening of intersheet packing, leading to the destabilization of the native state and subsequent access to the amyloidogenic intermediate states. On the other hand, Smith *et al.* (15) suggested that the I_T_ state is not obligatory for amyloid fibril formation based on real-time 2D NMR experiments. They did not observe any significant differences in structural features or the kinetics of the formation and deformation of the I_T_ state between the wild type and D76N. These findings indicated that the amyloidogenic process of D76N is distinct from that of the wild type.

In the present study, we investigated the pressure-induced unfolding of wild-type and D76N β2ms in order to elucidate the molecular properties of each state appearing in the folding process, particularly the amyloidogenic intermediate state. Previous studies investigated the effects of pressure on protein conformations and stabilities. Among the various denaturing perturbations, such as the denaturant, temperature, and pH, analyses of pressure-induced denaturation behavior provided us with unique information on protein molecules, *e.g.*, molar volume changes and thermal fluctuations (16, 17).

The results of our equilibrium unfolding experiment suggested that D76N unfolded at a lower pressure region than the wild type, as expected. Regarding folding kinetics, wild-type β2m showed a “roll-over”, *i.e.*, an increased refolding rate with pressure increases in the lower pressure range (less than 100 MPa), indicating the presence of transition states with a lower molecular volume than the preceding state. The results obtained were reasonably interpreted using a four-state folding model. The results of model fitting for the wild type revealed that conformational changes from the intermediate states to the native state underwent a transient volumetric reduction, indicating that the intermediate state had to unfold once and reassemble residue–residue interactions in order to acquire its native conformation. Furthermore, the I_T_ state of D76N showed enhanced hydrophobicity and accumulated more rapidly than the wild type in the folding process. Based on these results, we discussed the enhanced amyloidogenicity of the D76N mutant.

## Results

### pH dependence of the conformational stability of wild-type and D76N β2ms

The native state under physiological conditions was too stable to accomplish any pressure-induced unfolding within the accessible pressure range. Therefore, prior to the experiment for the pressure effect, we selected pH conditions under which the native conformation was slightly destabilized. Fig. S1*A* shows pH-dependent spectral changes in the wild type, which has already been published in our previous report (14). As we mentioned therein, the spectral peak was red-shifted with a simultaneous reduction in intensity when pH was decreased. Fig. S1*B* shows the pH-dependent <ν> values for wild-type β2m and D76N, where <ν> is center of spectral mass and indicates the averaged wavelength of the fluorescence spectrum (see Equation 3 in Experimental procedures). The wild type and D76N showed clear cooperative transitions (Fig. S1*B*, the red and green markers). We fit the data with sigmoidal curves to obtain the mid-point pH values of unfolding (pH_m_). The pH_m_ values obtained were 4.11 ± 0.04 and 4.54 ± 0.03 for the wild type and D76N, respectively. Based on these experiments, both the wild type and D76N assumed the native state at pH 6.0. Thus, in subsequent experiments, we prepared the sample solution at this pH.

### Equilibrium and kinetic measurements of pressure-induced unfolding

**Figure 2:**
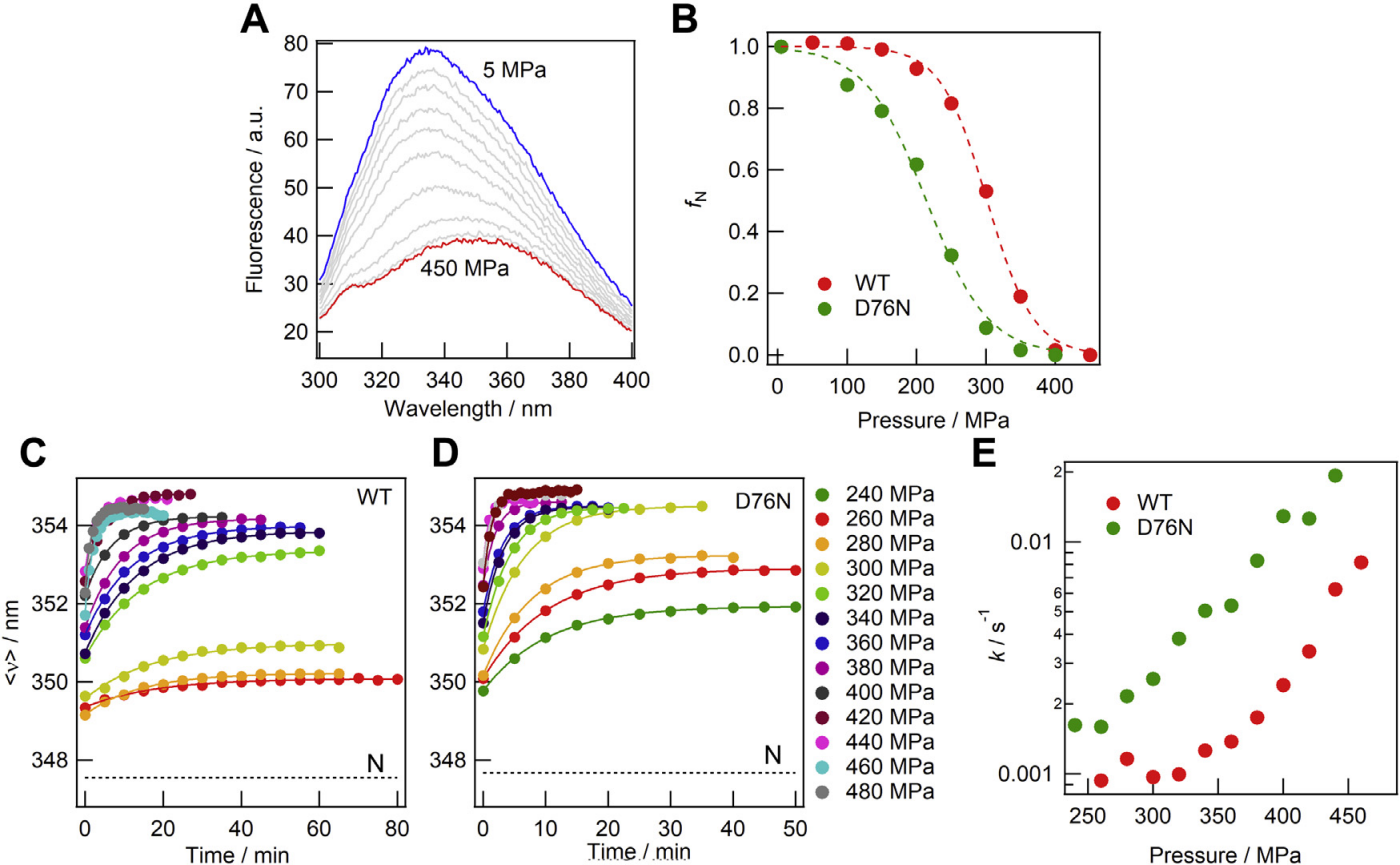
**Equilibrium and kinetic measurements of pressure-induced unfolding.***A*, pressure-dependent spectral changes in the tryptophan fluorescence of wild-type β2m. The *blue* and *red lines* indicate the spectra obtained at 5 and 450 MPa, respectively. These spectra were recorded 30 min after pressure was changed to the respective points. *B*, pressure dependence of the fraction of the native state based on the <ν> values for the wild type (*red*) and D76N (*green*). The *broken lines* are the theoretical curves based on the two-state unfolding model expressed by Equations 1 and 2 (see main text). *C* and *D*, the unfolding kinetics of wild-type (*C*) and D76N (*D*) β2ms probed through the time-dependent <ν> value. The *continuous lines* are single exponential curves fit to the data. The *dotted lines* indicate the <ν> value of the N state at 5 MPa before the unfolding reaction. *E*, plots of unfolding rate constants against the respective pressures for the wild type (*red*) and D76N (*green*).

Figure 2*A* shows the pressure dependence of the tryptophan fluorescence spectra of wild-type β2m at pH 6.0. These spectra were recorded 30 min after pressure was increased to the respective pressure points. Figure 2*B* shows the pressure dependence of the fraction of the native state based on the <ν> values for wild-type and D76N β2ms. Both species showed clear cooperative transitions. If there are only two states, namely the native (N) and unfolded (U) states, the free energy difference between these two state at respective pressure point (Δ*G*_NU_) is described by the following equation (16):

(1) $$\Delta G_{\text{ND}}=\Delta G_{0,\text{ND}}+P\Delta V_{\text{ND}}$$

where *P* is applied pressure, Δ*G*_0,NU_ is Δ*G*_NU_ at ambient pressure, and Δ*V*_NU_ is difference in molar volume between the N and U states. By assuming a simple two-state model, we fitted the fraction of the N state (*f*_N_) data for Δ*G*_0,NU_ and Δ*V*_NU_ using the following equation:

(2) $$f_{\text{N}}=1/\left(1+\exp\left(-\frac{\Delta G_{0,\text{ND}}+P\Delta V_{\text{ND}}}{RT}\right)\right)$$

The apparent values obtained were Δ*G*_0,NU_ = −22.5 ± 1.1 kJ/mol and Δ*V*_NU_ = 74.5 ± 3.6 ml/mol for the wild type and Δ*G*_0,NU_ = −12.0 ± 1.0 kJ/mol and Δ*V*_NU_ = 56.0 ± 4.6 ml/mol for D76N. It is noted that the obtained Δ*G*_0_ values agree very well with those reported by Mangione *et al.* (12) (−23.84 kJ/mol and −12.55 kJ/mol for the wild type and D76N, respectively).

We then measured unfolding kinetics. Immediately after the pressure had been increased to the desired point, repetitive measurements of spectra were initiated. The <ν> values of each spectrum were plotted against time (Fig. 2, *C* and *D*) and fit to a single exponential curve for rate constants. The rate constants obtained were plotted against the measurement pressure (Fig. 2*E*). According to previous findings, the time constants of the fast and slow phases were 0.1∼0.2 s and ∼800 s, respectively (8). Since the dead time of the present measurements was approximately ∼180 s, the present measurements only detected the slow phases. The unfolding rate constants for wild-type and D76N β2ms increased with pressure; however, the plot of the mutant shifted to a lower pressure range than that of the wild type (Fig. 2*E*).

Since the kinetic events observed were relatively slow, they were assumed to correspond to *cis* to *trans* isomerization reactions on the peptidyl–prolyl bond at Pro32 (8, 9, 12). In order to check this assumption, we also investigated the unfolding kinetics of ΔN6 and P32V β2ms, both of which are known to have the *trans* form of Pro32 in their native states and do not show a *cis*-*trans* conformational transition. As expected, these variants did not have a kinetic phase (Fig. S2), confirming that the slow kinetic phases observed in these unfolding experiments were caused by the *cis* to *trans* isomerization of Pro32.

### Kinetic measurements of refolding from the pressure-induced unfolded state

**Figure 3:**
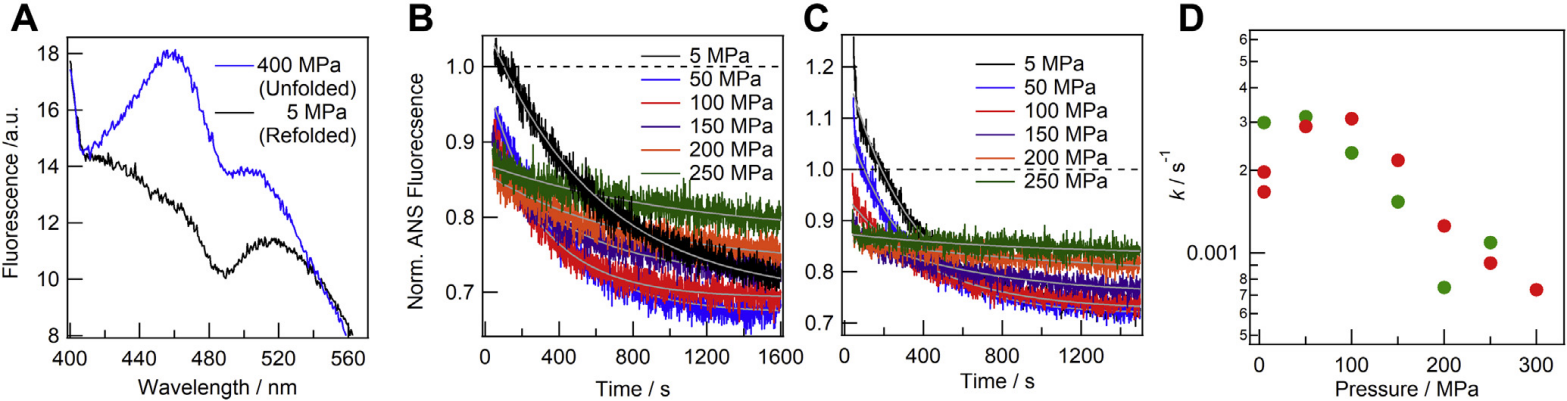
**Kinetic measurements of refolding from the pressure-induced unfolded state.***A*, ANS fluorescence spectra of the pressure-induced unfolded state (*blue*) and refolded state from the unfolded state (*black*) of the wild type are shown. *B* and *C*, refolding kinetics monitored by ANS fluorescence for the wild type (*B*) and D76N (*C*) are shown, where intensity was normalized with respect to the initial (unfolded state) intensity of the ANS fluorescence. The *white lines* are single exponential curves fit for rate constants. *D*, the pressure-dependent rate constants obtained for the wild type (*red*) and D76N (*green*) are shown.

We also attempted to investigate refolding kinetics by monitoring time-dependent <ν>; however, apparent kinetics were found to be accomplished within several minutes (Fig. S3) possibly because the fast phase accompanies the shift in <ν>, while the slow phase did not show significant changes in <ν>. Thus, instead of <ν>, we examined 1-anillino-8-naphthalene sulfonate (ANS) fluorescence. Figure 3*A* shows the spectra of the native state (black) and pressure-induced unfolded state (blue) of the wild type, indicating that the unfolded state induced the enhancement in ANS fluorescence, particularly at 460 nm. When ANS fluorescence at this wavelength was used to probe refolding kinetics, we found that it was able to monitor the refolding kinetics of the slow steps (Fig. 3, *B* and *C*).

Observations of refolding kinetics from the pressure-induced unfolded state were started immediately after the release of pressure to the respective pressure points. The fluorescence intensities obtained were normalized with respect to the initial (unfolded state) intensity of the ANS fluorescence (Fig. 3, *B* and *C*). The intensity of ANS fluorescence immediately after the pressure release for D76N was larger than that of the wild type (Fig. 3*C*), indicating that the burst-phase species of D76N has a more hydrophobic nature. Kinetic data were then fit with a single exponential curve to obtain rate constants. Figure 3*D* shows the pressure-dependent rate constants for wild-type and D76N β2ms. It is noted that rate constants of ANS association and dissociation were reported to be 10^1^∼10^2^ s^−1^ (18, 19). Therefore, the observed rate constant, which was smaller than those of the association/dissociation rates by four order of magnitude, likely reflected the conformational change of β2ms. The pattern of the pressure-dependent rate constants of D76N shifted toward a lower pressure range than that of the wild type, similar to unfolding kinetics. At the lower pressure region, a “roll-over” was noted, namely refolding kinetics became faster as pressure increased. This result indicated that transition state(s) appearing during the folding pathway had a lower molar volume than the preceding state (see Equation 5).

### Model fitting

**Figure 4:**
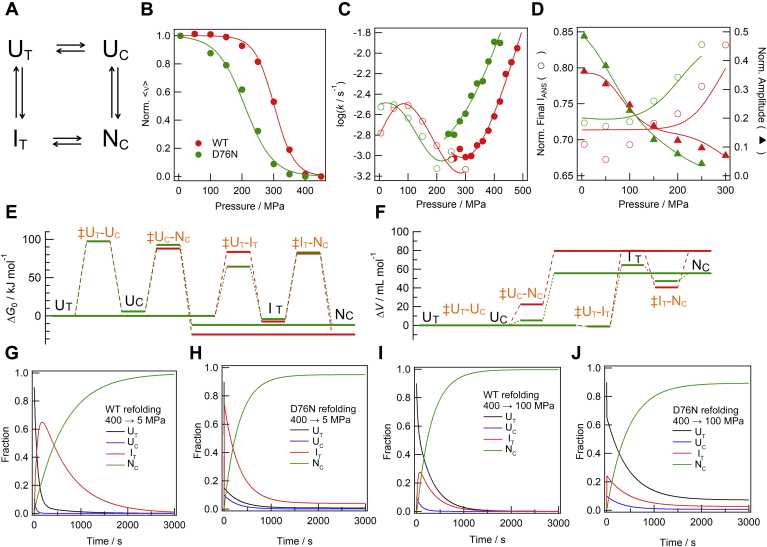
**Model fitting of experimental data.***A*, schematic presentation of the proposed four-state model. *B*–*D*, comparison of theoretical curves and experimental data for WT (*red*) and D76N (*green*). *B*, pressure dependence of the fraction of the native state. *C*, plots of refolding (*open circle*) and unfolding (*solid circle*) rate constants against the respective pressures. *D*, the pressure-dependent final intensity of ANS fluorescence (*open circle*) and exponential amplitude (*solid triangle*) obtained from the refolding experiments are shown. In panels *B*–*D*, the *continuous lines* are theoretical curves derived from the global fitting of the equilibrium (*B*) and kinetic (*C* and *D*) data to the four-state model (see Experimental procedures). Thus, although the data in *B* is the same as those in Figure 2*B*, the theoretical curve is different from that in Figure 2*B*. *E* and *F*, Δ*G*_0_ and Δ*V* diagrams obtained for WT (*red*) and D76N (*green*). *G*–*J*, calculated time-dependent populations of each species during refolding upon 400→5 MPa change for WT (*G*) and D76N (*H*), and those upon 400→100 MPa change for WT (*I*) and D76N (*J*).

We performed a fitting analysis with the kinetic data of pressure-induced unfolding and refolding experiments. Based on previous reports (8, 12), the four-state folding model shown in Figure 4*A* was assumed as the supposed model. The *G*_0_ and *V* values for the U_T_ state were set to be 0 as the reference state. Then, we assumed the Δ*G*_0_ and Δ*V* values for other stable states (U_C_, I_T_, and N_C_) as the differences between respective states and the U_T_ state. The Δ*G*_0_ and Δ*V* values for transition states between these four states (‡U_T_-U_C_, ‡U_C_-N_C_, ‡U_T_-I_T_, and ‡I_T_-N_C_) with respect to the U_T_ state were also introduced. In addition, normalized values of <ν> and intensity of ANS fluorescence (I_ANS_) for individual species were introduced as fitting parameters. Thus, there were 22 thermodynamic parameters (the Δ*G*_0_ and Δ*V* values for the U_T_ state were set to be 0 as the reference state as indicated by a in Table 1). The equilibrium and kinetic data were globally fitted to the theoretical curves with the fitting parameters (see Experimental procedures). However, since the number of the fitting parameters were too many and the kinetic data obtained in the present study were limited to “slow phases” according to previous studies, all of the parameters cannot be unambiguously identified. Thus, we introduced a couple of assumptions for this fitting. (i) The *cis-trans* kinetics in unfolded states (U_T_-U_C_) were fixed and invariable with respect to pressure (assuming that Δ*V* of U_T_, U_C_, and ‡U_T_-U_C_ are 0). Fixed values following by (i) are indicated by b in Table 1. (ii) During the fitting analysis, normalized I_ANS_ for individual species was set variable, whereas normalized <ν> was fixed to 0 for U_C_ and U_T_ and to 1 for I_T_ and N_C_. Δ*G*_0_ and Δ*V* values between the U_T_ and N_C_ states were based on those obtained in the equilibrium experiments performed above. Under these assumptions, experimental data displayed in Figures 4, *B*–*D* were simultaneously subjected to the global fitting. These data were successfully fitted with the parameters listed in Table 1. It is noted that, although the data in Figure 4*B* is basically the same as those in Figure 2*B*, the theoretical curve was that derived from the global fitting results. Figure 4, *E* and *F* show graphical representation of Δ*G*_0_ diagram at the ambient pressure and Δ*V* diagrams in a reaction coordinate form based on the parameters listed in Table 1, respectively. The Δ*G* diagrams at respective pressures were also calculated and displayed in Fig. S4. The time courses of the populations of these states upon refolding at the ambient pressure (Fig. 4, *G* and *H*) and at 100 MPa (Fig. 4, *I* and *J*) were also calculated. The refolding/unfolding time courses at respective pressures are also displayed in Fig. S4. (For the calculation process, see Supporting information).

**Table 1:** Fitting parameters obtained for pressure-dependent unfolding for WT and D76N β2m

β2m	Stable states	Δ*G*_0_/kJ mol^−1^	Δ*V*/ml mol^−1^	Norm. I_ANS_	Transition states	Δ*G*_0_/kJ mol^−1^	Δ*V*/ml mol^−1^
WT	U_T_	0^a^	0^a^	0.86 ± 0.02	‡U_T_-U_C_	97.6^b^	0^b^
D76N		0^a^	0^a^	0.85 ± 0.04		97.6^b^	0^b^
WT	U_C_	5.8^b^	0^b^	0.93 ± 0.06	‡U_C_-N_C_	88.0 ± 1.5	22.4 ± 5.4
D76N		5.8^b^	0^b^	0.85 ± 0.13		92.9 ± 1.0	5.2 ± 5.0
WT	I_T_	−7.4 ± 1.4	79.6 ± 3.8	1.08 ± 0.09	‡U_T_-I_T_	83.6 ± 3.7	−1.1 ± 0.5
D76N		−4.0 ± 0.03	64.3 ± 0.3	1.34 ± 0.07		64.4 ± 0.9	−1.2 ± 0.0
WT	N_C_	−24.2 ± 1.2	79.4 ± 3.8^b^	0.71 ± 0.01	‡I_T_-N_C_	81.4 ± 1.4	40.6 ± 5.8
D76N		−11.8 ± 0.6	55.6 ± 3.0^b^	0.70 ± 0.02		82.9 ± 0.2	47.2 ± 0.4

The upper and lower values in a cell are of WT and D76N β2ms, respectively.

a Set to be 0 as the reference state.

b Fixed following by assumption (i) (see text).

‡ Transition states between the indicated states.

## Discussion

### The overview of the obtained pressure-dependent folding/unfolding of β2m

**Figure 5:**
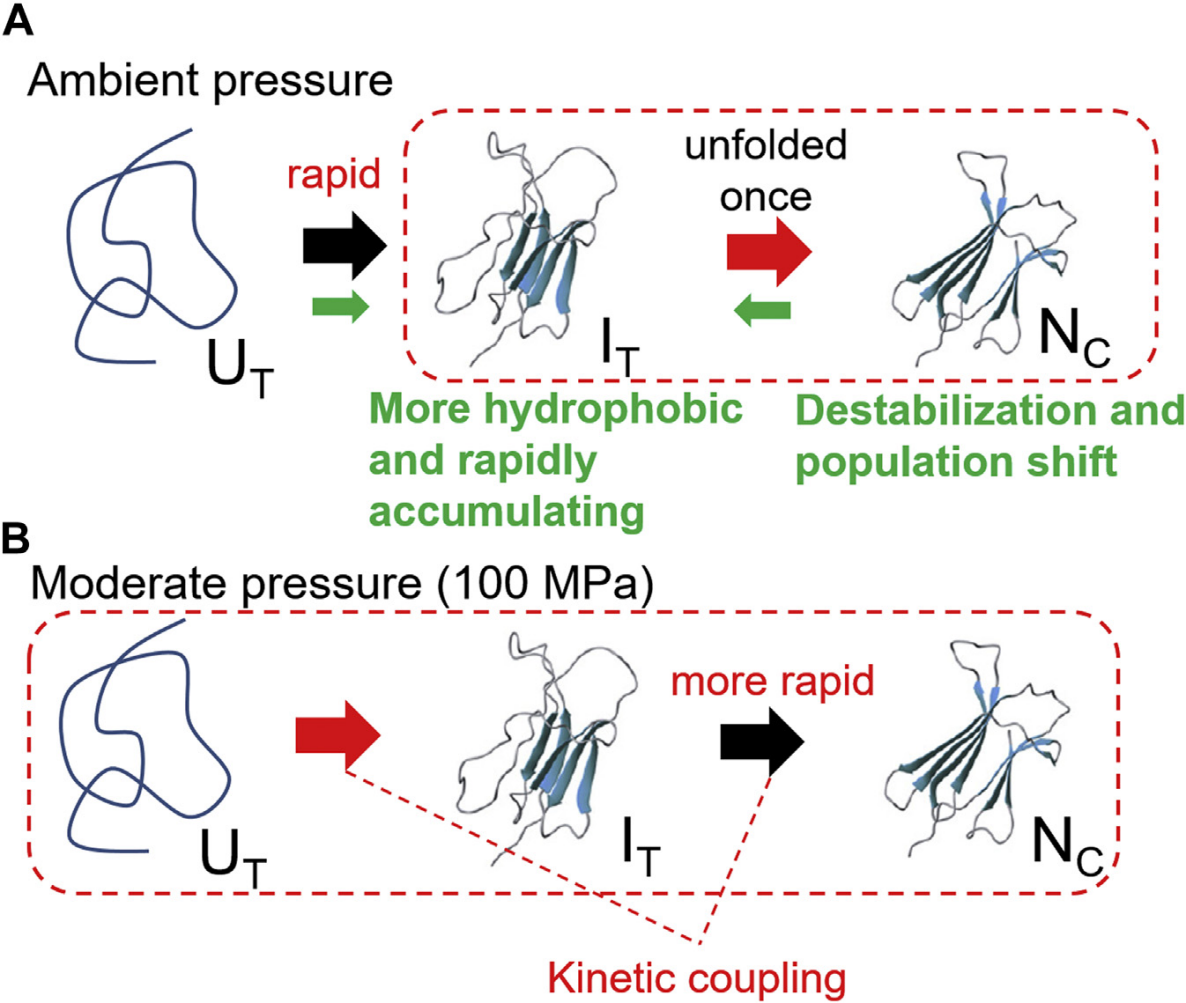
**Representation of species appearing in the folding process of β2m at ambient pressure (*A*) and at moderate pressure (100 MPa) (*B*).** The descriptions in *green* in (*A*) indicate the effects of the D76N mutation. The descriptions in *red* indicate the effects of pressure. The *red arrows* indicate the rate-limiting steps at respective pressures. The *broken lines* indicate the microscopic steps, which associated with the observable slow phases at respective pressures. Especially, the kinetic coupling with both the first and second steps occurred at around 100 MPa (*B*).

The equilibrium and kinetic data obtained in the present study were explained by the four-state model shown in Figure 4*A*, which is based on several models suggested by previous folding studies on β2m (8, 9, 12). Although the present data were limited to the slow phases, the obtained fitting parameters based on the model were converged fairly well with several assumptions (Fig. 4 and Table 1). The Δ*G*_0_ diagram obtained was consistent with that suggested by Sakata *et al.* (8) We focused our interest on the I_T_ state in the U_T_ → I_T_ →N_C_ pathway. As the *cis*-*trans* isomerization of the Pro32 is slow, the observed slow kinetics is derived from the second step (I_T_ →N_C_). Although there are no direct data of the fast phases, we found that the kinetic rates of the first and second steps of the U_T_ → I_T_ →N_C_ pathway were mutually dependent, and we were able to draw information about both phases from the observed slow phase (Fig. 5): Our analytical results told us that the rate-limiting steps was the second step (I_T_ →N_C_) at lower pressures (Figs. 4, *G* and *H* and 5*A*, and Fig. S4, *A*–*C*), whereas it changed to the first step (U_T_ → I_T_) at higher pressure (100 MPa) (Figs. 4, *I* and *J* and 5*B*, and Fig. S4, *E* and *F*). In the latter situation, the observed kinetic data was derived from the merged kinetics of the first and second steps and includes information of both steps. Such a phenomenon is called as “kinetic coupling of unfolding/refolding and prolyl isomerization” (8). In addition to the kinetic coupling, the observation of the pressure-dependent shift of the rate-limiting step enabled the model fitting giving plausible Δ*G*_0_ and Δ*V* values for each state on the basis of Equation 5 (Fig. 4, *E* and *F* and Table 1).

The estimated fluorescence intensities and evolutions of the population in each state explain the pressure dependences of the burst-phase change in the ANS fluorescence (Fig. 3, *B* and *C*): The burst-phase fluorescence was enhanced under the ambient pressure (especially in the D76N case), because the I_T_ state accumulates within the burst phase (Figs. 4, *G* and *H* and 5*A*). However, at 100 MPa, the accumulation of the I_T_ state becomes less (Figs. 4, *I* and *J* and 5*B*) because the activation energy between the I_T_ state and N_C_ state becomes lower relative to the I_T_ state (Fig. S4*D*). In such a situation, the folding apparently proceeds from the U_T_ state directly to N_C_ state and the burst-phase ANS fluorescence became weakened.

### Characteristics of I_T_-N_C_ step in the β2m refolding

The present results provide Δ*V* values for individual states. The Δ*V* value contains important structural information, based on which the native and intermediate states in the folding process were discussed.

The molecular volume change upon unfolding (Δ*V*_tot_) is the sum of three contributions: Δ*V*_tot_ =Δ*V*_vdW_ +Δ*V*_void_ +Δ*V*_hyd_, where Δ*V*_vdW_, Δ*V*_void_, and Δ*V*_hyd_ are the change in the van der Waals volume, void volume, and hydration volume upon unfolding, respectively. Royer *et al.* suggested that the main determinant of Δ*V*_tot_ is decrease in Δ*V*_void_ or the disappearance of the void space, the inner space of the protein molecule inaccessible by solvent molecules (20, 21). Chen and Makhatadze (22) additionally suggested the importance of the exposure of hydrophobic residues, which increases Δ*V*_tot_ *via* the large positive contribution of Δ*V*_hyd_ because water is excluded from the hydrophobic surface. Δ*V*_tot_ upon unfolding is generally negative because the former contribution (negative Δ*V*_void_) is slightly larger than the latter (positive Δ*V*_hyd_).

The present results told us a characteristic property of the folding process of β2m. It was found that the molar volume of I_T_ was comparable with that of N_C_, and the transition states between I_T_ and N_C_ (‡I_T_-N_C_) had a smaller molar volume than the previous (I_T_) and next (N_C_) states (Fig. 4*F*). These properties led to increases in the refolding rate at the lower pressure region for the wild type (Fig. 4*C*). This is in contrast to a standard folding case, in which the molar volume of the transition state is larger than the unfolded state and smaller than the folded state. According to the discussion mentioned above, a decrease in volume was caused by either a reduction in the hydrophobic surface or void volume; however, the latter likely explains the present results. The I_T_ state may assume an almost similar conformation to N_C_ but the local structures around Pro32 are frustrated (15, 23). Therefore, the packing of the I_T_ state is not as strict as the native N_C_ state, which results in an enhanced molecular volume. Packing deficiency and subsequent increase in molar volume due to formed void spaces were also reported in the amyloid fibril formation (24, 25, 26). Along with this interpretation, the I_T_ state may assume once unfolded conformations, in which the formed voids disappear, to proceed to the final N_C_ state. This may be a type of trapped state, and local unfolding is needed to reassemble these regions (Fig. 5*A*, the second step).

### Difference of ΔV between wild-type and D76N β2ms

Δ*V*_NC-UT_ values were found to be 79.4 and 55.6 ml/mol for the wild type and D76N, respectively, indicating that the increase in molar volume upon the formation of the native conformation of D76N β2m was 23.8 ml/mol smaller than that of the wild type. This difference can be also explained by the following two reasons. One is the difference in Δ*V*_void_: Due to the similarity in crystal structures between wild-type and D76N β2ms (11), this difference may not be apparently attributed to Δ*V*_void_. However, our previous report suggested that the wild type has larger flexibility in the native conformation than D76N (14), suggesting that the enhanced flexibility may increase the volume of the native state and subsequently contribute to the larger Δ*V*_tot_. Another possibility is the change in the Δ*V*_hyd_ contribution: The D76N mutation locally decreased the electrostatic potential, leading to the conversion of a hydrophilic surface to a hydrophobic surface. According to Chen and Makhatadze (22), Δ*V*_hyd_ is determined using the following equation: Δ*V*_hyd_ = *k*_NP_Δ*MSA*_NP_ + *k*_Pol_Δ*MSA*_Pol_, where *MSA*_NP_ and *MSA*_Pol_ are the molecular surface areas (MSA) of hydrophobic and hydrophilic regions, respectively. *k*_NP_ and *k*_Pol_ are the respective contributions of a given type of MSA to the hydration volume. If the reported values are used (*k*_NP_ = 0.38 Å and *k*_Pol_ = 0.03 Å), the Δ*V*_hyd_ of 23.8 ml/mol corresponds to a hydrophilic-to-hydrophobic conversion area of 113 Å^2^ since 23.8 ml/mol = *N*_A_ × (0.38 Å × 113 Å^2^ − 0.03 Å × 113 Å^2^), where *N*_A_ is Avogadro number. The accessible surface area of the side chain of the free aspartic acid is estimated to be 90 Å^2^ (27). Thus, although it may be overestimated, a one-residue replacement may cause significant change in Δ*V*_NU_. At present, the degree of contributions of these two possibilities to the difference in Δ*V*_tot_ is not clear, which warrants further study.

### Difference in the property of I_T_ state between wild-type and D76N β2ms

We observed the enhanced ANS fluorescence of burst-phase species. Figure 4, *G* and *H* shows the back-calculated time-dependent populations of respective species, showing that the burst-phase species is the I_T_ state. Fitting results quantitatively indicated that the intensity of the ANS fluorescence of D76N (1.34 ± 0.07) was significantly larger than that of the wild type (1.08 ± 0.09) (Table 1). Thus, the I_T_ state of D76N has a more hydrophobic nature. The Δ*V* of I_T_ was larger than N_C_ for D76N, whereas the Δ*V* of I_T_ was similar to that of N_C_ for the wild type (Fig. 4*F*), which can be also attributed to the larger hydrophobic surface area according to the discussion above. This interpretation is also consistent with the MD findings reported by Chong *et al.* (13). They suggested that the I_T_ state of D76N has a more hydrophobic surface area.

In addition, the fitting results showed that the D76N mutation affected the Δ*G*_0_ values of the native, intermediate, and transition states. Important finding was that the energy level of ‡I_T_-N_C_ for D76N was significantly lowered than that for the wild type (Fig. 4*E*), which made the accumulation of the I_T_ state from the unfolded state more rapid. Figure 4, *G* and *H* also show that the I_T_ state for D76N accumulated and reached to about 80% immediately after the initiation of refolding, indicating that almost all U_T_ species immediately converted to the I_T_ state. On the other hand, that of the wild type did slowly. It hit a maximum around 65% in population ∼100 s after the initiation of the refolding. On the other hand, the rate of the conversion from the I_T_ to the N_C_ states was not affected significantly by the mutation.

This is partly consistent with the results of Smith *et al.* (15): They monitored the I_T_ to the N_C_ conversion by using real-time 2D NMR measurements and found there was no significant change in the conversion rate. The transient population of the I_T_ state suggested by them was significantly lower than our results: They reported that the fraction of the I_T_ state immediately after refolding was approximately 3% for both the wild type and D76N on the basis of the NMR signal intensity. However, since the I_T_ state is likely more fluctuating and dynamic than the native state, the signal intensity was supposed to be weakened due to peak broadenings, leading to underestimation of the transient population of the intermediate states.

Based on the discussion above, the mechanism underlying the enhanced amyloidogenicity of D76N appeared to be the enhanced hydrophobicity of the I_T_ state and rapid accumulation of this state from the unfolded state (Fig. 5*A*). The reported specific interactions between D76N and a chaperone protein, α-crystalline, in the physiological conditions likely support this suggestion because such partially unfolded states are the main targets of chaperones (12, 28). In addition, the destabilization of the native state also contributed to increases in the accessibility of the amyloidogenic precursor state. Such alteration is likely significant if the surface/interface unfolding is a trigger of the protein aggregation (29): When unfolded on the membrane or tissue in the body, D76N is easier to form the pathogenic intermediate state than the wild type. Such a property can explain the changes in amyloidogenicity in *in vitro* experiments and the *in vivo* preference of tissue for deposition from collagen-rich joint areas to visceral regions in the body, resulting in different clinical symptoms (11, 15). The stabilization of the native state will be an effective approach for preventing amyloid fibril formation, similar to transthyretin amyloid fibril formation (30).

## Conclusion

We herein investigated the pressure-induced folding/unfolding of D76N β2m. We demonstrated that the folding process may be represented by a four-state model. The main difference between the wild type and D76N was the activation energy between the unfolded and the I_T_ states, leading to a more rapid accumulation of I_T_ state for D76N than that of the wild type. Furthermore, the I_T_ state of the D76N mutant had a more hydrophobic nature. These changes resulted in differences in pathological processes under physiological conditions.

## Experimental procedures

### Expression and purification of wild-type β2m and its variants

pAED4 plasmids harboring the wild-type and ΔN6, P32V, and D76N human β2m genes were used for protein expression (14). These β2m variants were expressed in *Escherichia coli* strain BL21 (DE3) (Novagen) and purified as previously described (31). We used M9 medium for protein expression.

### Pressure-induced unfolding equilibrium monitored by fluorescence

To monitor the conformational stability of proteins as a function of pressure, we used a high-pressure fluorescence spectrometer system incorporating a customized high-pressure optical vessel and inner optical cell (Syn-Corporation Co, Ltd) with an FP-6500 spectrofluorometer (JASCO Inc) (32, 33). The tryptophan fluorescence spectra of wild-type and D76N β2ms were collected from 5 to 450 MPa in 20 mM phosphate buffer (pH 6.0), 100 mM NaCl, and 25 °C. We applied 5 MPa instead of 0.1 MPa to avoid any effect from air bubbles in the inner optical cell. The excitation wavelength was set at 280 nm with a slit width of 5 nm, while the emission wavelength was 300 to 400 nm with a slit width of 5 nm. The spectra obtained were quantified using the center of the spectral mass, <ν>, which is defined by the following equation:

(3) $$\overset{<\nu >}{\underset{\nu_{init}}{\int }}Fd\nu =\frac{1}{2}\overset{\nu_{end}}{\underset{\nu_{init}}{\int }}Fd\nu$$

where ν_init_ and ν_end_ are the initial and end wavelengths of the spectra, and *F* is fluorescence intensity at respective wavelengths. Fluorescence intensities as well as spectral shapes were apt to vary from sample to sample probably because the orientation of the high-pressure cell was changed upon sample solution exchange. Thus, we recorded spectra of complete N state at ambient pressure and complete U state at 400 MPa as reference for each setup before the measurements and normalized with the reference spectra.

### Refolding and unfolding kinetics at various pressures monitored by fluorescence

To monitor unfolding kinetics, time-dependent changes in the tryptophan spectrum were measured. Immediately after pressure was increased to the desired value, tryptophan spectra were repeatedly recorded. The <ν> values for each spectrum were calculated and plotted against time. The measurement dead time was approximately 3 min due to manual pumping.

To monitor refolding kinetics, time-dependent ANS fluorescence changes were monitored. The sample solution was the same as that for unfolding measurements, except for the addition of 10 μM ANS. After the unfolding transition was equilibrated at 400 MPa, the ANS fluorescence spectrum was measured. Pressure was then lowered to the desired value and the continuous measurement of ANS fluorescence at 460 nm with a slit width of 10 nm was initiated. The excitation wavelength was 350 nm with a slit width of 5 nm and the measurement dead time was approximately 90 s. After the time-course measurement, the ANS fluorescence spectrum of the refolded state was also acquired.

### Analysis of kinetic data

We proposed the four-state model shown in Figure 4*A*. Differential equations for the time-dependent populations of each species were as follows:

(4) $$\frac{\text{d}}{\text{d}t}\left(\begin{array}{l} \left[\text{A}\right]\\ \begin{array}{l} \left[\text{B}\right]\\ \left[\text{C}\right] \end{array}\\ \left[\text{D}\right] \end{array}\right)=\left(\begin{array}{llll} -k_{\text{AB}}-k_{\text{AC}} & k_{\text{BA}} & k_{\text{CA}} & 0\\ k_{\text{AB}} & -k_{\text{BA}}-k_{\text{BD}} & 0 & k_{\text{DB}}\\ k_{\text{AC}} & 0 & k_{\text{CA}}-k_{\text{CD}} & k_{\text{DC}}\\ 0 & k_{\text{BD}} & k_{\text{CD}} & -k_{\text{DB}}-k_{\text{DC}} \end{array}\right)\left(\begin{array}{l} \left[\text{A}\right]\\ \begin{array}{l} \left[\text{B}\right]\\ \left[\text{C}\right] \end{array}\\ \left[\text{D}\right] \end{array}\right)$$

where A, B, C, and D represent the U_T_, U_C_, I_T_, and N_C_ states, respectively. The elements of the 4×4 matrix are microscopic rate constants for each elementary step and were calculated from Δ*G*_0_ and Δ*V* values at the respective pressure points, *e.g.*, *k*_AB_ is the microscopic rate constant of the U_T_→U_C_ reaction and was calculated with the following equation:

(5) $$k_{\text{AB}}=\frac{k_{\text{B}}T}{h}\exp\left(-\frac{\left(\Delta G_{0,\text{AB}}^{‡}-\Delta G_{0,\text{A}}\right)-P\left(\Delta V_{\text{AB}}^{‡}-\Delta V_{\text{A}}\right)}{RT}\right)$$

where *k*_B_, *h*, *R*, *T*, and *P* are the Boltzmann constant, Planck constant, gas constant, temperature, and pressure, respectively. Δ*G*^‡^_0,AB_ and Δ*V*^‡^_AB_ are the Gibbs free energy change and molar volume change of the transition state ‡U_T_-U_C_, respectively. Solving the differential equations, we obtained the time courses of the populations of individual states. The process of calculations for the time courses of the populations of individual states was explained in Supporting Information. From the time-dependent populations obtained, the <ν> value and the ANS fluorescence intensity were calculated as follows:

(6) $${<\nu >}_{\text{cal}}=\sum {<\nu >}_{\text{x}}\cdot \left[\text{x}\right]\left(t\right)$$

(7) $${\text{I}}_{\text{ANS},\text{cal}}=\sum {\text{I}}_{\text{ANS},\text{x}}\cdot \left[\text{x}\right]\left(t\right)$$

where x indicates the individual states (*i.e.*, A∼D), <ν>_x_ and I_ANS,x_ were the <ν> value and the ANS fluorescence intensities, respectively, for individual states. Fitting the calculated curves to a single exponential curve, apparent parameters, final intensity (values at *t*=∞), exponential amplitude, and *k* (rate constant), were obtained. The calculations of the theoretical curves and model fitting were performed with Igor Pro (Wavemetrics).

## Data availability

Data shared upon request. Send inquiries to Kazumasa Sakurai (sakurai@waka.kindai.ac.jp).

## Conflict of interest

The authors declare that they have no conflicts of interest with the contents of this article.

## References

[1] Iwata K., Matsuura T., Sakurai K., Nakagawa A., Goto Y. (2007). High-resolution crystal structure of β_2_-microglobulin formed at pH 7.0. J. Biochem..

[2] Naiki H., Hashimoto N., Suzuki S., Kiumura H., Nakakuki K., Gejyo F. (1997). Establishment of a kinetic model of dialysis-related amyloid fibril extension *in vitro*. Amyloid.

[3] Gejyo F., Yamada T., Odani S., Nakagawa Y., Arakawa M., Kunitomo T., Kataoka H., Suzuki M., Hirasawa Y., Shirahama T., Cohen A.S., Schmid K. (1985). A new form of amyloid protein associated with chronic hemodialysis was identified as beta 2-microglobulin. Biochem. Biophys. Res. Commun..

[4] Kad N.M., Myers S.L., Smith D.P., Smith D.A., Radford S.E., Thomson N.H. (2003). Hierarchical assembly of beta2-microglobulin amyloid *in vitro* revealed by atomic force microscopy. J. Mol. Biol..

[5] Corazza A., Pettirossi F., Viglino P., Verdone G., Garcia J., Dumy P., Giorgetti S., Mangione P., Raimondi S., Stoppini M., Bellotti V., Esposito G. (2004). Properties of some variants of human beta2-microglobulin and amyloidogenesis. J. Biol. Chem..

[6] Hoshino M., Katou H., Hagihara Y., Hasegawa K., Naiki H., Goto Y. (2002). Mapping the core of the β_2_-microglobulin amyloid fibril by H/D exchange. Nat. Struct. Biol..

[7] Yamaguchi K., Katou H., Hoshino M., Hasegawa K., Naiki H., Goto Y. (2004). Core and heterogeneity of beta2-microglobulin amyloid fibrils as revealed by H/D exchange. J. Mol. Biol..

[8] Sakata M., Chatani E., Kameda A., Sakurai K., Naiki H., Goto Y. (2008). Kinetic coupling of folding and prolyl isomerization of beta2-microglobulin studied by mutational analysis. J. Mol. Biol..

[9] Jahn T.R., Parker M.J., Homans S.W., Radford S.E. (2006). Amyloid formation under physiological conditions proceeds via a native-like folding intermediate. Nat. Struct. Mol. Biol..

[10] Chiti F., De Lorenzi E., Grossi S., Mangione P., Giorgetti S., Caccialanza G., Dobson C.M., Merlini G., Ramponi G., Bellotti V. (2001). A partially structured species of beta 2-microglobulin is significantly populated under physiological conditions and involved in fibrillogenesis. J. Biol. Chem..

[11] Valleix S., Gillmore J.D., Bridoux F., Mangione P.P., Dogan A., Nedelec B., Boimard M., Touchard G., Goujon J.M., Lacombe C., Lozeron P., Adams D., Lacroix C., Maisonobe T., Plante-Bordeneuve V. (2012). Hereditary systemic amyloidosis due to Asp76Asn variant β_2_-microglobulin. N. Engl. J. Med..

[12] Mangione P.P., Esposito G., Relini A., Raimondi S., Porcari R., Giorgetti S., Corazza A., Fogolari F., Penco A., Goto Y., Lee Y.H., Yagi H., Cecconi C., Naqvi M.M., Gillmore J.D. (2013). Structure, folding dynamics, and amyloidogenesis of D76N β2-microglobulin: Roles of shear flow, hydrophobic surfaces, and α-crystallin. J. Biol. Chem..

[13] Chong S.H., Hong J., Lim S., Cho S., Lee J., Ham S. (2015). Structural and thermodynamic characteristics of amyloidogenic intermediates of β-2-microglobulin. Sci. Rep..

[14] Sakurai K., Tomiyama R., Shiraki T., Yonezawa Y. (2019). Loosening of side-chain packing associated with perturbations in peripheral dynamics induced by the D76N mutation of β2-microglobulin revealed by pressure-NMR and molecular dynamic simulations. Biomolecules.

[15] Smith H.I., Guthertz N., Cawood E.E., Maya-Martinez R., Breeze A.L., Radford S.E. (2020). The role of the I_T_-state in D76N β2-microglobulin amyloid assembly: A crucial intermediate or an innocuous bystander?. J. Biol. Chem..

[16] Akasaka K. (2006). Probing conformational fluctuation of proteins by pressure perturbation. Chem. Rev..

[17] Kitahara R., Hata K., Li H., Williamson M.P., Akasaka K. (2013). Pressure-induced chemical shifts as probes for conformational fluctuations in proteins. Prog. Nucl. Magn. Reson. Spectrosc..

[18] Cattoni D.I., Kaufman S.B., Gonzalez Flecha F.L. (2009). Kinetics and thermodynamics of the interaction of 1-anilino-naphthalene-8-sulfonate with proteins. Biochim. Biophys. Acta.

[19] Chou W.Y., Bieber C., Matthews K.S. (1989). Tryptophan and 8-anilino-1-naphthalenesulfonate compete for binding to trp repressor. J. Biol. Chem..

[20] Roche J., Caro J.A., Norberto D.R., Barthe P., Roumestand C., Schlessman J.L., Garcia A.E., Garcia-Moreno B.E., Royer C.A. (2012). Cavities determine the pressure unfolding of proteins. Proc. Natl. Acad. Sci. U. S. A..

[21] Rouget J.B., Aksel T., Roche J., Saldana J.L., Garcia A.E., Barrick D., Royer C.A. (2011). Size and sequence and the volume change of protein folding. J. Am. Chem. Soc..

[22] Chen C.R., Makhatadze G.I. (2017). Molecular determinant of the effects of hydrostatic pressure on protein folding stability. Nat. Commun..

[23] Kameda A., Hoshino M., Higurashi T., Takahashi S., Naiki H., Goto Y. (2005). Nuclear magnetic resonance characterization of the refolding intermediate of beta2-microglobulin trapped by non-native prolyl peptide bond. J. Mol. Biol..

[24] Chatani E., Kato M., Kawai T., Naiki H., Goto Y. (2005). Main-chain dominated amyloid structures demonstrated by the effect of high pressure. J. Mol. Biol..

[25] Iadanza M.G., Silvers R., Boardman J., Smith H.I., Karamanos T.K., Debelouchina G.T., Su Y., Griffin R.G., Ranson N.A., Radford S.E. (2018). The structure of a beta2-microglobulin fibril suggests a molecular basis for its amyloid polymorphism. Nat. Commun..

[26] Lee Y.H., Chatani E., Sasahara K., Naiki H., Goto Y. (2009). A comprehensive model for packing and hydration for amyloid fibrils of beta2-microglobulin. J. Biol. Chem..

[27] Tien M.Z., Meyer A.G., Sydykova D.K., Spielman S.J., Wilke C.O. (2013). Maximum allowed solvent accessibilites of residues in proteins. PLoS One.

[28] Natalello A., Mangione P.P., Giorgetti S., Porcari R., Marchese L., Zorzoli I., Relini A., Ami D., Faravelli G., Valli M., Stoppini M., Doglia S.M., Bellotti V., Raimondi S. (2016). Co-fibrillogenesis of wild-type and D76N beta2-microglobulin: The crucial role of fibrillar seeds. J. Biol. Chem..

[29] Hasegawa K., Ozawa D., Ookoshi T., Naiki H. (2013). Surface-bound basement membrane components accelerate amyloid-beta peptide nucleation in air-free wells: An *in vitro* model of cerebral amyloid angiopathy. Biochim. Biophys. Acta.

[30] Bulawa C.E., Connelly S., Devit M., Wang L., Weigel C., Fleming J.A., Packman J., Powers E.T., Wiseman R.L., Foss T.R., Wilson I.A., Kelly J.W., Labaudiniere R. (2012). Tafamidis, a potent and selective transthyretin kinetic stabilizer that inhibits the amyloid cascade. Proc. Natl. Acad. Sci. U. S. A..

[31] Chiba T., Hagihara Y., Higurashi T., Hasegawa K., Naiki H., Goto Y. (2003). Amyloid fibril formation in the context of full-length protein: Effects of proline mutations on the amyloid fibril formation of beta2-microglobulin. J. Biol. Chem..

[32] Maeno A., Matsuo H., Akasaka K. (2013). Tyrosine/tyrosinate fluorescence at 700 MPa: A pressure unfolding study of chicken ovomucoid at pH 12. Biophys. Chem..

[33] Maeno A., Matsuo H., Akasaka K. (2009). The pressure-temperature phase diagram of hen lysozyme at low pH. Biophysics.

