# Supplementary material for: Enhanced accessibility and hydrophobicity of amyloidogenic intermediates of the β2-microglobulin D76N mutant revealed by high-pressure experiments
Source: J Biol Chem. 2021 Jan 26;296:100333. doi: 10.1016/j.jbc.2021.100333 (PMC7950326; doi:10.1016/j.jbc.2021.100333)
Supplement: Supplemental Figures S1–S4 [file mmc1.pdf]

Supporting Information for

Enhanced accessibility and hydrophobicity of amyloidogenic intermediates of the β2-microglobulin D76N mutant revealed by high-pressure experiments

Kazumasa Sakurai[1,2,*] and Ryosuke Tomiyama[2]

[1]High Pressure Protein Research Center, Institute of Advanced Technology, Kindai University, 930 Nishimitani, Kinokawa, Wakayama 649-6493, Japan.

[2]Department of Biotechnology, Faculty of Biology-oriented Science and Technology, Kindai University, Wakayama 649-6493, Japan

*To whom correspondence should be addressed.

E-mail: sakurai@waka.kindai.ac.jp; phone: +81-736-77-0345 (ex. 5004)

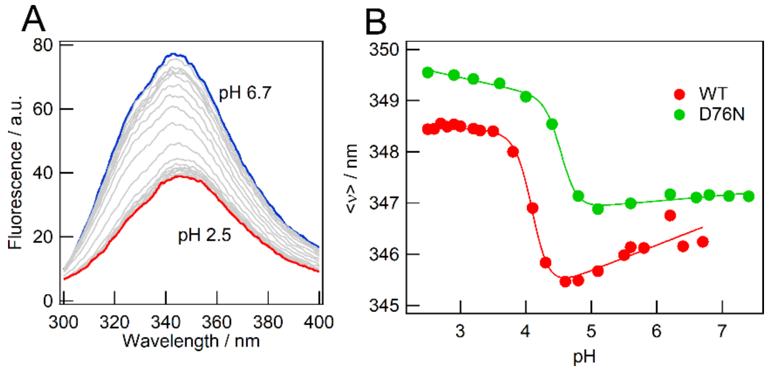

Figure S1. pH titration experiments. (A) pH-dependent spectral changes in the tryptophan fluorescence of wild-type β2m. The blue and red lines indicate the spectra obtained at pH 7.5 (starting point) and 2.5 (end point) of the titration, respectively. (B) pH-dependent <ν> values for wild-type (red) and D76N β2m (green), where <ν> is center of spectral mass and indicates the averaged wavelength of the fluorescence spectrum (see Eq. 3 in Experimental procedures in the main text). The continuous lines indicate sigmoidal curves fit for the midpoint pH of the unfolding reactions. This figure is basically the same as that we previously reported.(1)

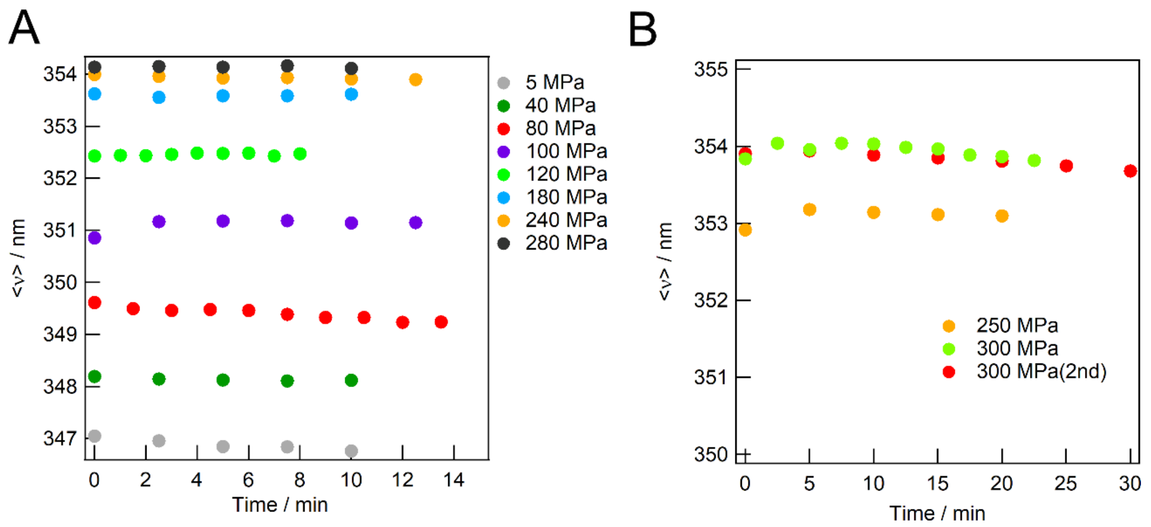

Figure S2. Time-dependent changes in <ν> values immediately after an increase in pressure from the ambient pressure to the indicated values for ΔN6 (A) and P32V (B), the refolding of which did not include slow *cis-trans* conformational changes. These variants did not show significant changes in the <ν> value, confirming that the slow folding process corresponded to *cis-trans* conformational changes.

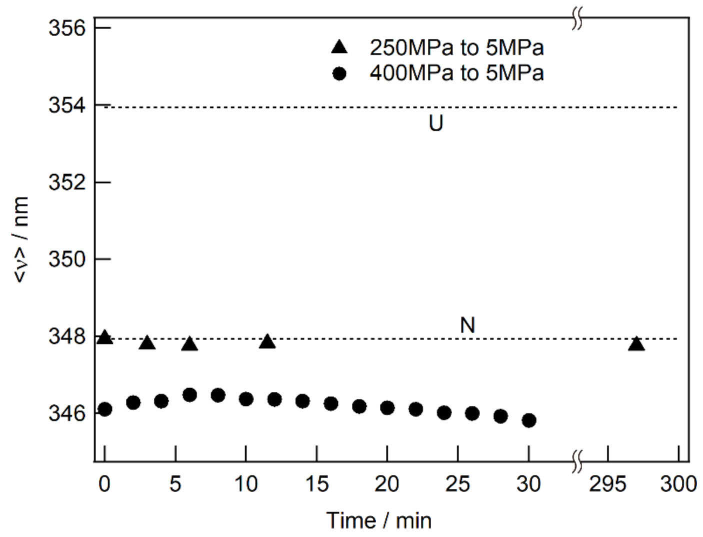

Figure S3. Time-dependent changes in <ν> values of wild-type β2m immediately after a decrease in pressure from values indicated to ambient pressure. The dotted lines indicate the <ν> values for the native (N) and unfolded (U) states. Wild-type β2m showed a similar <ν> value to that of the N state within the dead time, and no significant change was subsequently observed. These results indicate that the intermediate state after the fast phase already showed a similar <ν> value to the N state and also that the slow *cis-trans* phase did not accompany a significant change in the <ν> value.

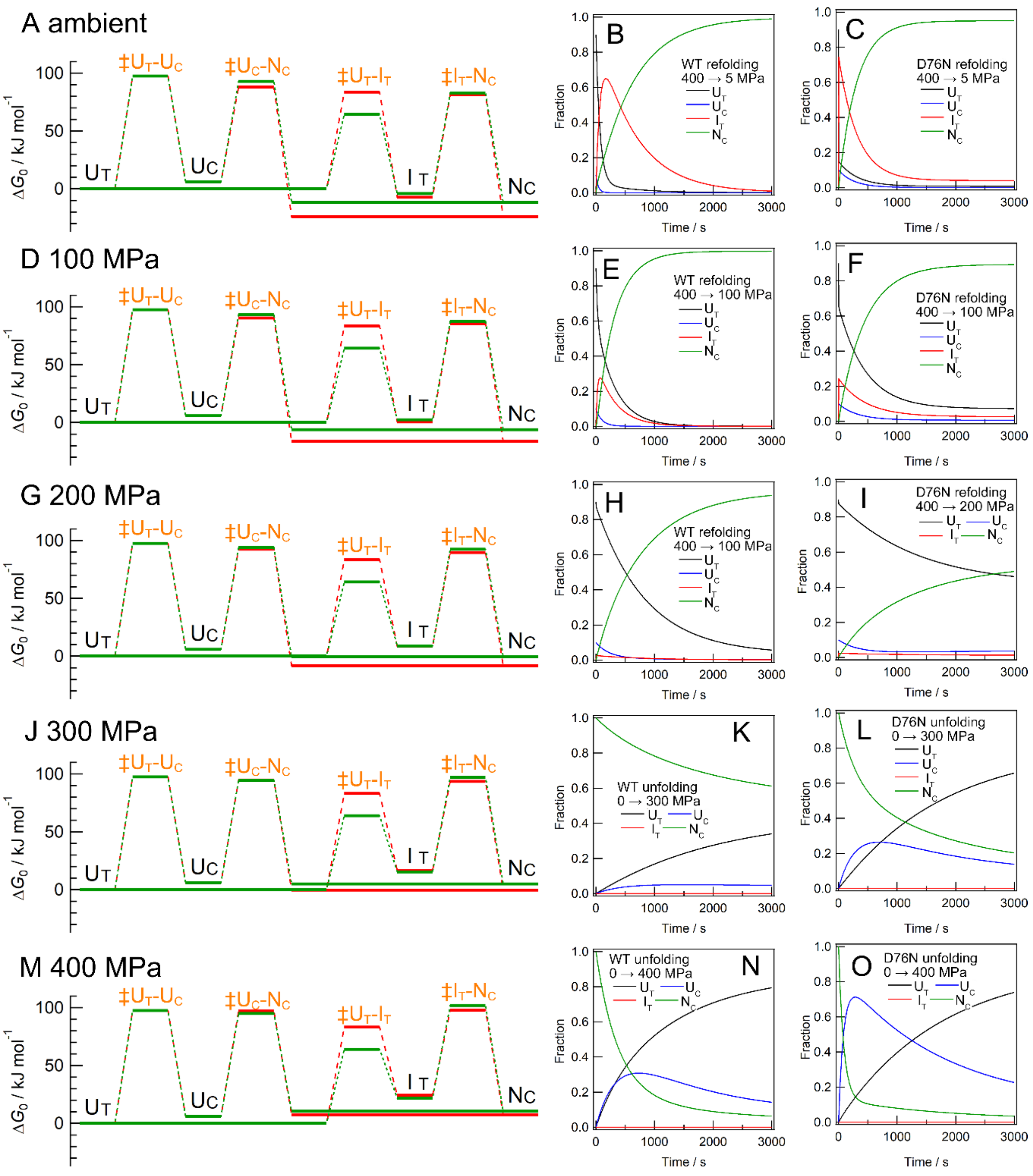

Figure S4. The results of the model fitting of the pressure-dependent kinetic data. (left column) Δ*G* diagrams obtained for WT (red) and D76N (green) at the ambient pressure (A), 100 MPa (D), 200

MPa (G), 300 MPa (J), 400 MPa (M). (central and right column) Calculated time-dependent populations of each species during refolding or unfolding upon the pressure change described in each panel. Figures 4E, G, H, I, J in the main text are the same as panels A, B, C, E, F in this figure, respectively, for a clear illustration of pressure dependences of Δ*G* diagrams and time-dependent populations of each species.

Supplementary Experimental procedure

pH-dependent conformational changes monitored by tryptophan fluorescence

Sample conditions were as follows: 0.05 mg ml^{-1} protein, 2 mM sodium phosphate, 2 mM sodium acetate, and 100 mM NaCl at 25°C. The excitation wavelength was set at 280 nm with a slit width of 5 nm, while the emission wavelength was 300-400 nm with a slit width of 5 nm. The starting pH was 7.5. The pH of 3 mL of the sample solution in a 1 cm×1 cm quartz cell was gradually reduced by the addition of 1 N HCl and spectra were obtained with a FP-6500 spectrofluorometer at respective pH points. pH-dependent $\langle \nu \rangle$ values were analyzed with a sigmoid function to obtain the midpoint pH value of unfolding. pH-dependent $\langle \nu \rangle$ values were analyzed with a sigmoid function to obtain the midpoint pH value of unfolding.

The calculation process of the time-dependent population of each state

We proposed a 4-state model shown in Figure 3a in the main text. The differential equations for the time-dependent populations of each species were shown as follows:

$$\frac{\mathrm{d}}{\mathrm{d}t}\begin{pmatrix}[\mathrm{A}]\\ [\mathrm{B}]\\ [\mathrm{C}]\\ [\mathrm{D}]\end{pmatrix} = \begin{pmatrix}-k_{\mathrm{AB}}-k_{\mathrm{AC}} & k_{\mathrm{BA}} & k_{\mathrm{CA}} & 0\\ k_{\mathrm{AB}} & -k_{\mathrm{BA}}-k_{\mathrm{BD}} & 0 & k_{\mathrm{DB}}\\ k_{\mathrm{AC}} & 0 & -k_{\mathrm{CA}}-k_{\mathrm{CD}} & k_{\mathrm{DC}}\\ 0 & k_{\mathrm{BD}} & k_{\mathrm{CD}} & -k_{\mathrm{DB}}-k_{\mathrm{DC}}\end{pmatrix}\begin{pmatrix}[\mathrm{A}]\\ [\mathrm{B}]\\ [\mathrm{C}]\\ [\mathrm{D}]\end{pmatrix}$$

(Eq. 4, see the main text)

where A, B, C, and D represent the U_T, U_C, I_T, and N_C states, respectively. The vertical vector containing the populations of each species ([A], [B], [C], and [D]) is represented as **p**. The 4×4 matrix is represented as **K**. The elements of **K** are microscopic rate constants for each elementary step and were calculated from ΔG_0 and ΔV values at respective pressure points, *e.g.*, k_{AB} is the microscopic rate constant of the $U_T \rightarrow U_C$ reaction and was calculated with the following equation:

$$k_{\mathrm{AB}} = \frac{k_{\mathrm{B}}T}{h}\exp\left(-\frac{\left(\Delta G^{\ddagger}_{0,\mathrm{AB}}-\Delta G_{0,\mathrm{A}}\right)-P\left(\Delta V^{\ddagger}_{\mathrm{AB}}-\Delta V_{\mathrm{A}}\right)}{RT}\right) \quad \text{(Eq. 5, see the main text)}$$

After diagonalization, **K** is converted to $\mathbf{U}^{-1}\mathbf{\Sigma}\mathbf{U}$, where **U** is a unitary matrix and **Σ** is a diagonal matrix containing eigenvalues of **K**, which are apparent rate constants and represented as λ, as diagonal elements. Equation 4 is then modified as follows:

$$\frac{\mathrm{d}}{\mathrm{d}t}\mathbf{p} = \mathbf{K}\mathbf{p} = \mathbf{U}^{-1}\mathbf{\Sigma}\mathbf{U}\mathbf{p} \quad \text{(Eq. 4')} \quad \rightarrow \quad \frac{\mathrm{d}}{\mathrm{d}t}\mathbf{U}\mathbf{p} = \mathbf{\Sigma}\mathbf{U}\mathbf{p} \quad \text{(Eq. S1)}$$

If **p'** is defined as **Up** (**p'** = **Up**) and contains converted populations of each species, [A'], [B'], [C'], and [D'], Eq. (S1) is rewritten as follows:

$$\frac{\mathrm{d}}{\mathrm{d}t}\begin{pmatrix}[\mathrm{A}']\\ [\mathrm{B}']\\ [\mathrm{C}']\\ [\mathrm{D}']\end{pmatrix} = \begin{pmatrix}-\lambda_1 & 0 & 0 & 0\\ 0 & -\lambda_2 & 0 & 0\\ 0 & 0 & -\lambda_3 & 0\\ 0 & 0 & 0 & -\lambda_4\end{pmatrix}\begin{pmatrix}[\mathrm{A}']\\ [\mathrm{B}']\\ [\mathrm{C}']\\ [\mathrm{D}']\end{pmatrix} \quad \text{(Eq. S1')}$$

λ_4 is practically 0 because the rank of **K** is 3 at most. The differential equations of Equation S1' are then solved as follows:

$$\mathbf{p}' = \begin{pmatrix}[\mathrm{A}']\\ [\mathrm{B}']\\ [\mathrm{C}']\\ [\mathrm{D}']\end{pmatrix} = \begin{pmatrix}[\mathrm{A}'_0]\exp(-\lambda_1 t)\\ [\mathrm{B}'_0]\exp(-\lambda_2 t)\\ [\mathrm{C}'_0]\exp(-\lambda_3 t)\\ [\mathrm{D}'_0]\exp(-\lambda_4 t)\end{pmatrix} \quad \text{(Eq. S2)}$$

The populations of the original species ($\mathbf{p}$) are back-calculated to be obtained as follows:

$$\mathbf{p} = \begin{pmatrix} [\mathrm{A}](t) \\ [\mathrm{B}](t) \\ [\mathrm{C}](t) \\ [\mathrm{D}](t) \end{pmatrix} = \mathbf{U}^{-1} \begin{pmatrix} [\mathrm{A}'_0] \exp(-\lambda_1 t) \\ [\mathrm{B}'_0] \exp(-\lambda_2 t) \\ [\mathrm{C}'_0] \exp(-\lambda_3 t) \\ [\mathrm{D}'_0] \exp(-\lambda_4 t) \end{pmatrix} \quad \text{(Eq. S3)}$$

The populations of the original species were numerically calculated using Equation S3 with the ΔG_0 and ΔV values listed in Table 1. These calculations, including determinations of the eigenvalues of $\mathbf{K}$ and making unitary matrices, were performed with Igor Pro (Wavemetrics, Lake Oswego, OR).

References

1. Sakurai, K., Tomiyama, R., Shiraki, T., and Yonezawa, Y. (2019) Loosening of Side-Chain Packing Associated with Perturbations in Peripheral Dynamics Induced by the D76N Mutation of β2-Microglobulin Revealed by Pressure-NMR and Molecular Dynamic Simulations. *Biomolecules* **9**, 491
